# The introduction of a mandatory mask policy was associated with significantly reduced COVID-19 cases in a major metropolitan city

**DOI:** 10.1371/journal.pone.0253510

**Published:** 2021-07-21

**Authors:** Nick Scott, Allan Saul, Tim Spelman, Mark Stoove, Alisa Pedrana, Alexander Saeri, Emily Grundy, Liam Smith, Michael Toole, Chandini Raina McIntyre, Brendan S. Crabb, Margaret Hellard

**Affiliations:** 1 The Burnet Institute, Melbourne, Australia; 2 Department of Epidemiology and Preventive Medicine, Monash University, Melbourne, Australia; 3 BehaviourWorks Australia, Monash Sustainable Development Institute, Monash University, Melbourne, Australia; 4 Biosecurity Program, The Kirby Institute, University of New South Wales, Sydney, Australia; 5 Department of Immunology and Pathology, Monash University, Melbourne, Australia; 6 Doherty Institute and School of Population and Global Health, University of Melbourne, Parkville, Australia; 7 Department of Infectious Diseases, The Alfred Hospital, Melbourne, Australia; Swiss Tropical & Public Health Institute, SWITZERLAND

## Abstract

**Background:**

Whilst evidence of use of face masks in reducing COVID-19 cases is increasing, the impact of mandatory use across a large population has been difficult to assess. Introduction of mandatory mask use on July 22, 2020 during a resurgence of COVID-19 in Melbourne, Australia created a situation that facilitated an assessment of the impact of the policy on the epidemic growth rate as its introduction occurred in the absence of other changes to restrictions.

**Methods and findings:**

Exponential epidemic growth or decay rates in daily COVID-19 diagnoses were estimated using a non-weighted linear regression of the natural logarithm of the daily cases against time, using a linear spline model with one knot (lspline package in R v 3.6.3). The model’s two linear segments pivot around the hinge day, on which the mask policy began to take effect, 8 days following the introduction of the policy. We used two forms of data to assess change in mask usage: images of people wearing masks in public places obtained from a major media outlet and population-based survey data. Potential confounding factors (including daily COVID-19 tests, number of COVID-19 cases among population subsets affected differentially by the mask policy–e.g., healthcare workers) were examined for their impact on the results. Daily cases fitted an exponential growth in the first log-linear segment (k = +0.042, s.e. = 0.007), and fitted an exponential decay in the second (k = -0.023, s.e. = 0.017) log-linear segment. Over a range of reported serial intervals for SARS-CoV-2 infection, these growth rates correspond to a 22–33% reduction in an effective reproduction ratio before and after mandatory mask use. Analysis of images of people in public spaces showed mask usage rose from approximately 43% to 97%. Analysis of survey data found that on the third day before policy introduction, 44% of participants reported “often” or “always” wearing a mask; on the fourth day after, 100% reported “always” doing so. No potentially confounding factors were associated with the observed change in growth rates.

**Conclusions:**

The mandatory mask use policy substantially increased public use of masks and was associated with a significant decline in new COVID-19 cases after introduction of the policy. This study strongly supports the use of masks for controlling epidemics in the broader community.

## Introduction

Australia, like many countries, experienced a rise in coronavirus disease (COVID-19) cases in early 2020, peaking on 28^th^ March 2020 and then declining in April after federal and state governments introduced strict community controls, travel bans and quarantining of international arrivals [1]. Throughout late June and July (i.e. the start of the Southern winter) there was a resurgence of COVID-19 cases confined to Australia’s second most populous state, Victoria, with 13,078 cases detected between 14^th^ June and 10^th^ August and new daily case numbers peaking at 686 on 5^th^ August. Almost all cases (95%) were in the state capital, Melbourne, a city of 4.93 million people.

The resurgence of COVID-19 in Victoria led to multiple control measures being reintroduced in urban Melbourne, and by the end of September, daily numbers had declined to fewer than 20 and are continuing to decline [2, 3]. The first major restrictions to be introduced were defined in Victoria as “Stage 3 “restrictions and consisted of:

- closure of pubs, bars, entertainment venues, places of worship, restricting restaurants and cafes to takeaway only, and limiting public gatherings to two people; and
- requiring people in the restricted area to stay home, with only four reasons to leave home: shopping for essentials, caregiving, exercise, and work or study that cannot be done from home).

These restrictions were introduced at 11:59pm on 1^st^ July 2020 in 10 postcodes (out of approximately 550 in Melbourne) that had 70% of the Victorian diagnosed COVID-19 cases at the time. The area covered was expanded on 4^th^ July to include two additional postcodes and complete quarantine/isolation of nine public housing tower blocks where residents were considered to be at high risk; residents were required to stay inside their homes under the ‘detention’ directives, until all residents in each tower had been tested for presence of SARS-CoV-2 and infected people put into isolation. Depending on the tower, this took up to one week.

On 8^th^ July, Stage 3 restrictions were extended to include all of metropolitan Melbourne and the adjoining Mitchell Shire (hereafter, “Melbourne”), and Victoria’s borders with other states/jurisdictions were closed (Table 1). Previous work has shown that the introduction of Stage 3 restrictions slowed but did not reverse the exponential growth in daily cases; the growth rate fell from 0.140 to 0.037 per day. This reduction was estimated to have averted 9,000–37,000 new infections throughout July, compared to a scenario of continued trend in growth.

**Table 1:** Directions issued by Victoria’s chief health officer in Melbourne and Mitchell Shire in July and August 2020.

Date:	Directions
July 8 at 11:59pm	Stage 3 restrictions introduced to all of metropolitan Melbourne and the adjoining Mitchell Shire (“Melbourne”)
July 20	Government announces that the mandatory use of masks or face coverings will be introduced in public settings for Melbourne on the July 22 at 11.59 pm
July 22 at 11:59pm	Mandatory use of masks or face coverings in public settings for Melbourne
August 2	8 pm curfews and 5 km restrictions on movement for metropolitan Melbourne (excluding Mitchell Shire)
August 5 at 11:59pm	Stage 4 restrictions for metropolitan Melbourne (excluding Mitchell Shire)

Mandatory use of masks or other face coverings including surgical masks, cloth masks, face shields, scarves and bandanas (hereafter, masks) in public settings was announced on the 20^th^ July. The order came into force in Melbourne on 22^nd^ July with existing Stage 3 restrictions remaining in place. With very few exceptions, the mandatory use covered all spaces outside the home, everybody 12 and older regardless of social distancing and was strongly enforced with fines of A$200 imposed for non-compliance.

This was followed by an 8pm curfew and 5km movement restrictions introduced on 2^nd^ August, and Stage 4 restrictions (defined in Victoria as closure of childcare and non-essential businesses, including retail, and schools delivering online learning only) on 5^th^ August (Table 1).

The World Health Organization (WHO), the United States’ (USA) Centre for Disease Control and Prevention (CDC), other public health organisations and governments recommend the use of masks in specific circumstances [4–10]. However, despite these recommendations [4, 11–18] there is limited data from community settings supporting mask use. A systematic review and meta-analysis has examined how physical distancing, use of face masks and eye protection affect the spread of COVID-19, SARS, and MERS in both community and healthcare settings [5]. This included 172 observational studies across 16 countries but did not identify any published randomised controlled trials on the effectiveness of masks. The review found that mask use (including respirator use) could result in an 85% risk protection overall, with surgical masks and well-designed cloth masks giving 67% protection, but only six studies included data on COVID-19 and only three were from non-healthcare settings (and all three assessed SARS infection). Additional studies have been published examining the impact mask use (mandatory or consensual) on the incidence of COVID-19 cases [8, 14, 19–25], but collectively they illustrate the challenges in estimating the effectiveness of masks in community settings; in all of these studies either the introduction of masks not mandatory (hence the uptake of masks was not an immediate change making impact difficult to measure), limited information is available about actual mask uptake, or masks were introduced at the same time as other control measures making it difficult to disentangle their impact.

Since this study was undertaken there have been further studies on the impact of masks. A recent CDC update reviewed these studies [26] listing cohort and population-based studies that showed a positive effect by the introduction of or use of masks. One study on the introduction of masks in Arizona paralleled the situation we describe in this paper: a change in covid-19 incidence following the state-wide introduction of masks [6]. although this paper does not document the speed at which the mask mandate was implemented nor the magnitude of the change in mask usage and the introduction of multiple other measures (closure of bars etc., soon after mask introduction) limiting the generalizability of the conclusions. However, of relevance to our Melbourne study was the observation that the apparent impact on covid-19 incidence lagged the introduction of masks by about 12 days.

In July 2020 in Melbourne, the introduction of mandatory masks was strongly enforced and well separated in time from the introduction of other control practices, and with the large and quantified change in mask usage. This provides an important opportunity to quantitatively compare the pre- and post-mask intervention epidemic growth and decay rates.

## Methods

### Analysis of COVID-19 cases 10^th^ July to 10^th^ August inclusive

Daily diagnosed COVID-19 case numbers in Victoria (excluding cases in returned international travellers in hotel quarantine) were derived from the cumulative cases reported in daily updates [2] from the Victorian Department of Health and Human Services (DHHS).

The growth or decay of the daily number of new cases was approximated using an exponential model.

This means that the logarithm of the daily cases should increase or decrease linearly with time. The growth or decay rates of the epidemic were estimated using a non-weighted linear regression to fit the natural logarithm of the daily cases against time, using a linear spline model with one knot [27]. The knot allows for a possible deflection in the line of best fit during the period of observation, dividing the model into two linear segments that are connected at a hinge day. The assumption of linearity of the two segments, and hence exponential growth/decay, was tested by assessing whether studentized residuals were normally distributed and independent of the time (i.e., no significant heteroskedasticity). Due to the generation interval for SARS-CoV-2 infection and delays in testing and reporting, there is an expected delay between the introduction of restrictions and observing them in diagnosis data. The hinge day was estimated to be 8 days following the introduction of masks, based on a previous report observing an 8 day delay from the introduction of Stage 3 restrictions in Melbourne and a change in the epidemic growth rate [28], biological plausibility (a mean generation interval estimated at 4–6 days [29, 30], combined with delays in test-seeking and reporting), and assessment of model robustness, with sensitivity analyses used to explore alternate assumptions (S1 File).

This analysis is similar to the approach used in a previously published study on the impact of “Stage 3” restrictions in Melbourne, that showed that the growth fitted two exponential segments and also established that the change in worth was consistent with a lag of 8 days. It differs from that study in using spline fit with a single knot rather than two separate regressions, since this provides a theoretically better model requiring the segments to share a common incidence on the day that the pre- and post-intervention growth constants diverge.

While this analysis estimated the instantaneous growth constant (*k*) (i.e., the slope of the ln(daily cases) vs time regression) for each segment, the difference between these growth constants was used as the primary measure to test for an association between masks and epidemic growth/decay rates.

We have chosen the 10^th^ July as the starting day for this study, based on previous work observed that the introduction of Stage 3 took eight days to have an observed impact on the growth rates of the daily case data [28]. Since the current study specifically examines the impact of addition of masks on transmission, to avoid including the impact of curfew (introduced 2nd August) or Stage 4 restrictions (introduced 5th August) [2] we terminated the study period 8 days after the introduction of the curfew (10th August). Thus, the study period was from 10^th^ July to 10^th^ August. The period prior to the mandatory introduction of masks on the 11.59pm on 22 July is referred to as the *pre-mask period* and the period from the 23^rd^ July as the *post-mask period*.

We calculated the doubling/halving time of the epidemic as *t*_2_ = ln(2)/*k*

We also calculated an approximate range of *R*_*eff*_ as the relative change in new cases over one serial interval for SARS-CoV-2 infections using the Lotka–Euler equation. Due to uncertainties in the serial interval, we estimate a range for R_eff_ based on two estimates: a normal distribution with mean 3.96 days and standard deviation 4.75 days based on 468 pairs of infections in China [29], and a gamma distribution with mean 6.49 days and standard deviation 4.90 days based on 1015 pairs of infections in China, Japan and Singapore [30]. We have included changes in estimated *R*_*eff*_ only as a secondary estimate to allow comparisons with other studies, but emphasize that the estimates of *R*_*eff*_ are subject to uncertainty in estimates of the generation interval (approximated as the serial interval) and its distribution.

### Photographic observation of mask use

We assessed changes in mask-wearing behaviours using images from the digital archive of *The Age* (https://www.theage.com.au/), one of the two major daily Victorian newspapers. To ensure we captured all images (not just those published), the librarian/digital archivist from the newspaper reviewed consecutive photos in their archive that were taken between July 10 and August 2, 2020 (14 days before and 14 days after mandatory mask policy introduction). From these, all photos taken in public locations in urban Melbourne, such as streetscapes and shopping centres, and contained clear images of people were extracted and used to calculate the proportion of people wearing masks in public (number of people wearing masks / number of people in the image) across three time periods: period 1, 10–19 July (the period preceding mandatory mask policy announcement); period 2, 20–22 July (period between announcing and commencing mandatory mask wearing); and period 3, 23 July to 2 August (mandatory mask wearing period). We excluded people who were potentially exempt from mask wearing under the relevant directions, such as children and people drinking or eating while in public. The proportions of individuals wearing masks versus not wearing masks in each period were compared using cross-sectional chi-square tests. The meta data associated with each photograph is listed in the Supplementary file “PhotoMetadata.xlsx”. The original photographs can be obtained using these metadata from *The Age* (contact details are on their website).

### The Survey of COVID-19 Responses to Understand Behaviour (SCRUB) project—analysis of mask use

The Survey of COVID-19 Responses to Understand Behaviour (SCRUB) project includes measures of protective behaviour adherence among Australians during the COVID-19 pandemic; it commenced in March 2020. The survey targets a representative (age, gender, metro/regional) sample of 1000 Victorian residents and 700 Australian residents outside Victoria in each round. This study was approved by the Monash University Human Research Ethics Committee (ID 23854). Participants completed and on-line consent form as part of the survey.

An item measuring self-reported mask use frequency was included from round 6 onwards. Here we include data that spanned the date of mask introduction from round 6 (data collected 20–26 July 2020) for the question “*In the past 7 days*, *how frequently have you taken the following actions*?*”* and the specific item “*Wear a face mask whenever in public” (response scale*: *Never*, *Rarely*, *Sometimes*, *Often*, *Always)*., These categories were used to plot the data but were collapsed into a dichotomous outcome to estimate adherence (*Never*, *Rarely*, *Sometimes* versus) *Often*, *Always*. Respondent postcode and survey completion date were used to identify responses from people living within Melbourne and the daily responses were tabulated.

### Possible confounders

Data collected to explore potential confounding effects included 1) daily cases from regional Victoria, 2) daily cases in health care workers, 3) daily COVID-19 tests, 4) the proportion of diagnosed cases assigned to a known cluster within 24 hours of testing, and 5) mobility indices for Melbourne residents.

Daily cases in regional Victoria were derived from the cumulative cases reported in daily updates. Daily cases in health care workers, which were classified as acquired in health care and aged care facilities, were obtained from the Victorian DHHS web site. Daily COVID-19 tests and the proportion of diagnosed cases found to be close contacts of known clusters (a cluster is two or more cases previously shown to be close contacts and assumed to have been infected from an initial index case) within 24 hours of testing. This was used as a surrogate to measure changing efficiency in contact tracing over time. The data were obtained from the Australian Broadcasting Corporation’s coronavirus summary [31]. Mobility indices for Melbourne were obtained from CityMapper. The Citymapper Mobility Index is calculated by comparing trips planned in the Citymapper app to an arbitrary reference usage period, to assess whether mobility (relative to the reference period) changed over time during the observation period. The reference period used was the four weeks between January 6 and February 2, 2020 (i.e., prior to COVID-19). A day is defined as midnight to midnight UTC, thus in Melbourne overlaps calendar days.

Mask use in residents of regional Victoria was not mandated and use in health care and aged care facilities in Melbourne may not have changed since they were in near universal use prior to the introduction of the policy. Therefore, we analysed the growth rates in regional Victoria and in health care workers (HCW) on the expectation that these should show a limited change in growth rates in the pre-and post-mask periods. The aged care data were not available for the whole period and were not analysed. We undertook secondary regression analyses using dependent variables of log (total daily cases less regional cases), log (total daily cases less HCW cases) to test if regional cases and HCW cases had a significant impact of the results obtained with the total Victorian cases.

We also used linear regression of potential confounders (number of tests, log (ratio of total daily cases to number of daily tests), proportion of cases assigned to a cluster and mobility index) as a function of time to determine if they changed significantly during the observation period.

Finally, we performed a secondary linear spline regression with one knot regression of the log of the Melbourne cases that included daily tests and mobility index as well as time as independent variables.

## Results

### Association of masks with a change in epidemic growth rate

There were 11,714 cases reported in Melbourne between 10^th^ July and 10^th^ August inclusive, with daily cases increasing from 143 cases on 10^th^ July 10 to a peak of 686 cases on 5^th^ August, before declining to 310 cases on 10^th^ August.

**Fig 1:**
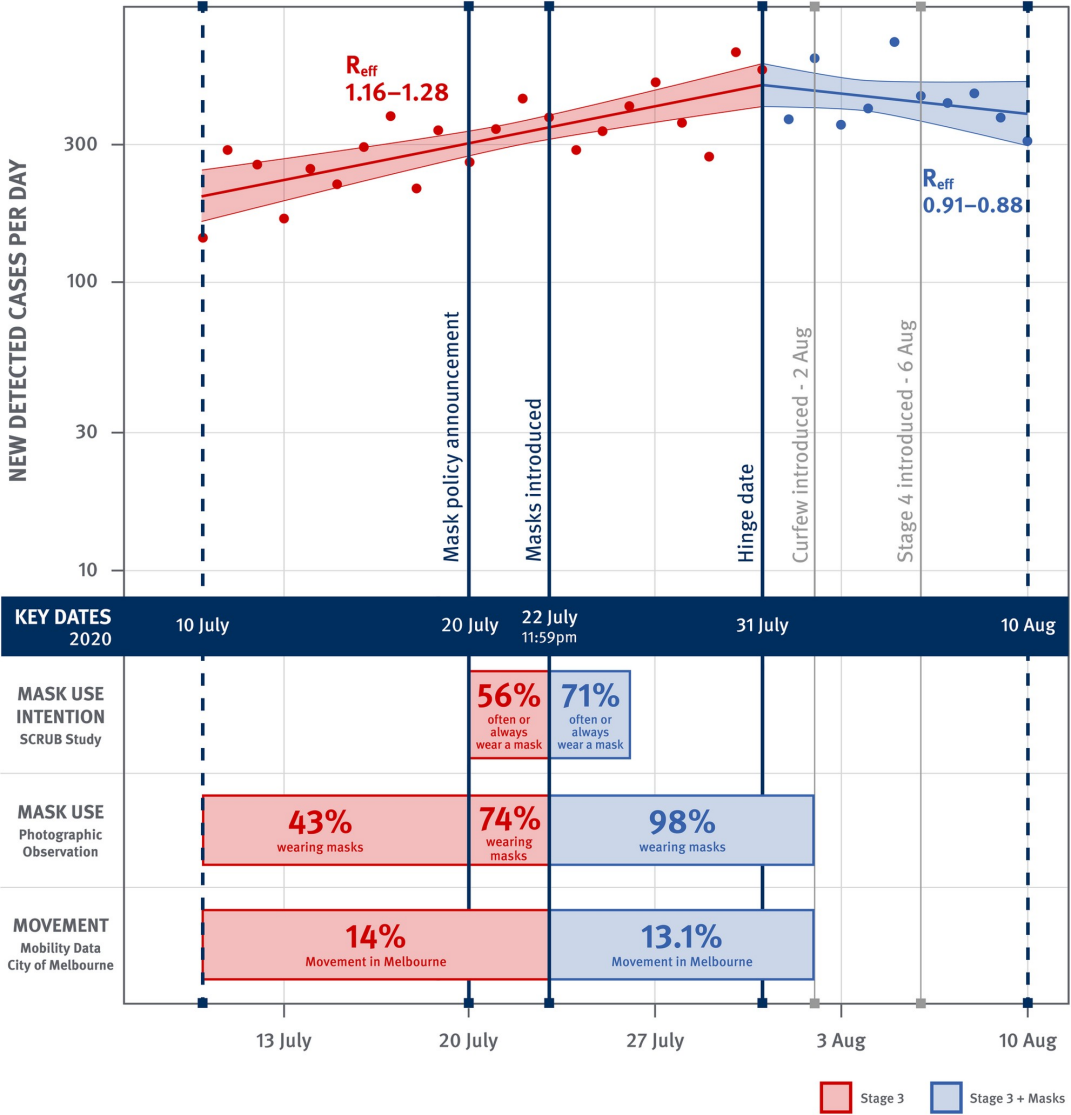
COVID-19 cases detected per day in Victoria. Observed daily cases (dots) and fitted linear spline model with a hinge day on 31st July, with shading representing upper and lower 95% confidence interval of the fitted curves. Red: analysis period for pre-masks; Blue: analysis period for post-masks.

The first log-transformed segment of the linear spline model, representing the pre-mask period, was consistent with an exponential growth in daily cases (*k* = +0.042, standard error [s.e.] = 0.007; p<0.001). This growth equated to a projected doubling of cases every 16.5 days (95% CI: 12 to 25 days). The second log-transformed linear segment, representing the post-mask period, was consistent with an exponential decay of daily cases (*k* = -0.023, s.e. = 0.017; p = 0.190). This decay equated to a projected halving of cases every 30 days (95% CI: doubling every 65 days to halving every 12 days). The difference in exponential growth/decay rates between the pre- and post-masks periods was highly statistically significant (Δ*k* = -0.065, s.e. = 0.022; p = 0.006) (Fig 1 and Table 2).

**Table 2:** Coefficients from the linear spline model with a knot on 31st July.

	Estimate	Std. Error	t value	Pr(>|t|)
Intercept	5.301	0.096	55.378	0.000
Slope estimate, pre-mask	0.042	0.007	5.858	0.000
Slope estimate, with masks	-0.023	0.017	-1.342	0.190
**Change in slope, introduction of mandatory masks**	**-0.065**	**0.022**	**-2.953**	**0.006**

Residual standard error: 0.239 on 29 degrees of freedom; Multiple R-squared: 0.582 Adjusted R-squared: 0.554; F-statistic: 20.21 on 2 and 29 DF, p-value: < 0.001.

These growth rates correspond to approximately a 22–33% reduction in *R*_*eff*_, depending on estimates of the serial interval for SARS-CoV-2. Using a mean serial interval of 3.96 days [29], this corresponds to a 22% decline in R_eff_ from 1.16 in the pre-mask period to 0.91 in the post-mask, while using a mean serial interval of 6.5 days [30], this corresponds to a 33% decline in R_eff_ from 1.29 to 0.86.

Fig 1 describes a longitudinal comparison of newly detected cases across time periods in a single population without explicit reference to a comparator population. This is distinct from a difference-in-difference approach which compares differences in outcomes across multiple time periods between a population of interest and a comparator or control group. Our analysis has no such comparator group.

On testing our model for goodness-of-fit, the distribution of studentized residual values of the log transformed cases was indistinguishable from a normal distribution and no evidence of heteroskedasticity (Breusch-Pagan test) (S1 File). Thus, the data was an excellent fit to an exponential increase in the Stage 3 period followed by an exponential decrease after masks became mandatory. Sensitivity analysis of the hinge day suggests that eight days following the introduction of masks had a high adjusted coefficient of determination R^2^ among other choices, and that the estimated change in slope would be greater for a longer lag time.

### Photographic observation of mask use

We analysed 44 published photographic images taken between July 10 and August 2, 2020. They included a range of public locations, including streetscapes, markets, train stations and supermarkets, and included 304 different individuals (after excluding those potentially exempt from mask use, i.e., children estimated to be under 12 and adults while they were eating or drinking). Nineteen photos taken in period 1 (prior to mandatory mask policy announcement) contained 101 different individuals, of whom 43 (43%) were wearing masks. Seven photos taken in period 2 (following mandatory mask policy announcement but prior to policy introduction) contained 47 different individuals, of whom 35 (74%) were wearing masks. Eighteen photos taken in period 3 (mandatory mask wearing period) contained 156 different individuals, of whom 153 (98%) were wearing masks. The proportion of individuals wearing masks significantly increased from period 1 to period 2 (*χ*^*2*^(1) = 13.09, *p*<0.001) and again from period 2 to period 3 (*χ*^*2*^(1) = 29.42, *p*<0.001). Photo metadata are listed in S1 Data.

### SCRUB study–self reported mask use

**Fig 2:**
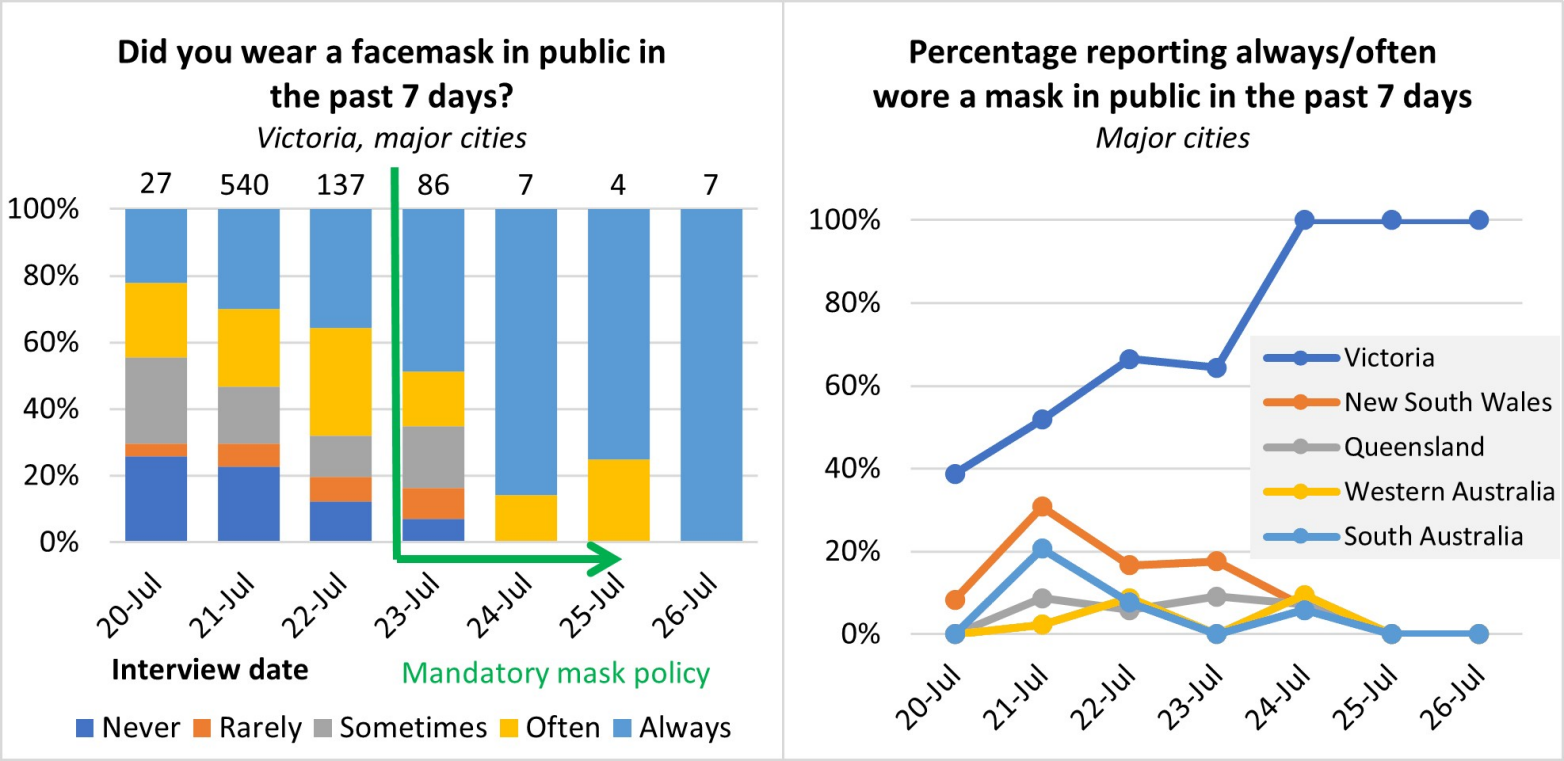
Results from the SCRUB survey. Left: for Round 6 respondents (20–26 July) in Victorian Stage 4 restriction areas, reported frequency of mask use in the past 7 days. Numbers on top of bars represent total respondents. Right: the percentage of respondents who reported always or often wearing a mask over time, Victorian Stage 4 restriction areas and compared to major cities in other states. The number of respondents varied from survey to survey and state to state but averaged 38.6 for each state and time point and the details are listed in S1 File.

Analysis of data from Round 6 of the SCRUB study found 44% of participants interviewed in Melbourne on the third day before the mask policy was introduced (July 20, 2020) reported they “often” or “always” wore a mask, and 100% of those interviewed on the fourth day afterwards (July 26) reported “always” wearing a mask. The survey found that 56% of participants reported “often” or “always” wearing a mask over 20–22 July, and 71% over 23–26 July (*χ*^*2*^(1, N = 808) = 8.73, *p* = 0.002) (Fig 2). This change in mask usage was not observed in SCRUB data collected from participants from other Australian states.

### Investigation of confounding variables

The proportion of non-metropolitan daily cases remained low (approximately 5%) across the period of interest (July 10 –August 10, 2020) and paralleled total Victorian cases. The proportion of daily cases in health care workers classified as acquired in health care and aged care facilities increased steadily, but there was no significant change in the case growth rates across the pre- and post-mask periods. Subtracting rural cases or cases in healthcare workers from the total cases made no significant difference to the calculated growth constants (S1 File).

There was a significant decrease in the number of tests per day performed over the study period, with approximately 33% fewer tests on the last day, 10^th^ August, than the first, 10^th^ July (linear regression: intercept 29,496; slope -310; *p* = 0.026). In addition, the pre- and post-mask period growth constants for the exponential increase and decrease in positive test ratio were higher than the corresponding growth rates for cases. However, the difference in the growth rates for pre- and post-mask were similar for cases and for the positive test ratio (S1 File). The proportion of cases that were assigned to known clusters within 24 hours was approximately constant throughout the analysis period.

The mobility data showed no significant change: 14.0% mobility pre-mask and 13.1% post-mask (p = 0.09) relative to mobility in February 2020, prior to the onset of the Australian COVID-19 epidemic (Fig 1).

Including daily tests and the mobility index as additional covariates in the multivariate linear regression made little difference to the estimated growth rates and neither the coefficient for mobility (-0.034, p = 0.28) nor tests (6 x 10−^6^, p = 0.36) were significantly different to zero (S1 File).

Changes to daily temperature could impact on virus transmissibility. However, data supplied by the Australian Bureau of Meteorology for Olympic Park in the centre of Melbourne over the observation period, failed to show any significant trend over time (S1 File).

## Discussion

We assessed growth rates in daily COVID-19 diagnoses in Victoria, Australia before and after the introduction of a mandatory mask policy in Melbourne (where 95% of Victoria’s diagnosed cases occurred), finding a highly significant decrease of 0.065 per day in the growth rate, from 0.042 per day to -0.023 per day. This decrease in growth rate is consistent with a change from an epidemic that is exponentially increasing with a doubling time of 16.5 days, to an epidemic that is exponentially decreasing with a halving time of 30 days. This also corresponds to with a change in the estimated pre-mask range of *R*_*eff*_ from about 1.16 to 1.28 (assuming a serial interval of 4 to 6.5 days) [29, 30] to a post-mask range of *R*_*eff*_ from about 0.91 to 0.88. The critical outcome is that introduction of masks was associated with a change from an increasing to a decreasing number of daily cases of COVID-19. This observation is consistent with modelling of the impact of mask usage. This model also predicts that usage of mask made the critical difference between the epidemic growing and contracting [32]. This took place in the context of existing stringent control measures and it is unclear what effect the masks would have had in the absence of these other measures. An important feature of this study is an observed, rapid and substantial increase in the use of masks within the population after 22^nd^ July 2020, as demonstrated in both self-report data and photographic data, that preceded a significant change in the growth rate of the epidemic. The rapid transition in the adoption of mask usage, based on photographic evidence, was striking: from 43% before the announcement of the upcoming mandatory mask policy, to an average of 74% in a three-day transition period between announcement and policy introduction, to near-complete compliance (98%) after policy implementation. The photographic data was complemented by self-report survey data that showed consistent rapid change, from just over 40% of participants reporting always or often wearing a mask on July 20, to 100% reporting always wearing a mask on 26 July (Fig 2).

### Methodological strengths and weaknesses

The introduction of masks on top of existing control program in Victoria provides an important guide important opportunity to inform public health policy [33]. The important strengths of this Melbourne study are

- Stable exponential growth in the daily cases prior to introduction of masks and no other interventions during the study period; and
- A major and measured change in mask usage to close to total coverage of 12 and older people in public areas; and
- High daily testing rates for SARS-CoV-2 infection (average of 370 per 100,000 in Victoria) and low positive test rates (average of 1.6% during the study), suggesting that most cases (although not all) would be detected.

We measured the overall impact associated with the introduction of mandatory mask use on daily cases. Care should be taken in ascribing causality. We cannot determine whether masks had a direct effect or whether near-universal adoption of masks reminded wearers to engage in other behaviours recommended to reduce SARS-CoV-2 transmission, including regular hand hygiene and physical distancing. Interestingly, introduction of masks in Melbourne coincided with a decrease in the growth rate in rural areas, where masks were not introduced, which is consistent with an indirect effect, a decrease in seeding of rural areas from the Melbourne, or both.

An assumption was needed regarding the time-lag between the implementation of a policy and observing its impact on detected cases. We used a time-lag of eight days after introduction of the mandatory mask policy, which is biologically plausible given the mean generation intervals reported and consistent with previous analyses in Melbourne and resulted in a model with a high goodness of fit.

Participants in the SCRUB Study were meant to report their mask use in the previous seven days, but the rapid change in behaviour over 20–26 July suggests participants interpreted this question as referring to their mask use on the day they responded to the survey. There were also only 18 responders in the final three days of the survey that again limits the precision of the post-mask estimate, but it does support the change in mask usage judged from the larger photographic survey. Subsequent surveys of 1313 Victorians (13 to 18 August) and 994 Victorians (31 Aug to 3 September) reported 93.6% and 93.4%, respectively, that they “always” used a mask.

Other factors may have contributed to the decline in SARS-CoV-2 transmission at the time the mandatory mask policy was introduced. Melburnians may have reduced their overall movement in response to other official messages or how they interacted with each other, such as whether people physically distanced from each other. Examination of mobility data across the relevant time period suggests mobility was stable [34, 35], although unpublished behavioural data collected through July suggests people were less likely to interact with other people as the month wore on, and when they did, kept physically distanced (*pers comm*., *J McCaw*, *Doherty Institute*). It is difficult to know whether this change in interaction and physical distancing would increase or decrease the effect we observed. The decrease in interactions/contact may also have affected case numbers but the decline in interactions was steady over time, so cannot account for the hinge effect we observed. Moreover, reduced interaction could mean that the mask policy was more effective than measured, because masks were still effective despite potentially fewer episodes of risky contact. Changes to the time needed for contact tracing may have influenced the result; if the time taken from identifying a case to contacting and isolating all their contacts fell substantially during the period in question, it may have created its own reduction in transmission. However, the proportion of cases assigned to a known cluster within 24 hours, and unpublished data from the Victorian DHHS (pers comm., B Sutton, DHHS Victoria), suggests the time taken to perform contact tracing was stable across the period of interest.

To make the analysis as robust as possible, we did not exclude health care workers classified as having acquired COVID-19 in health care and aged care facilities from the analysis, despite increasing numbers of cases in this group over the study period, reflecting the high occupational risk of infection in these settings. However, subtracting cases of health care workers from the Melbourne totals made little difference to the overall growth constants. Health care workers were wearing extensive personal protective equipment (PPE), including masks, in their work environment before mandatory mask policy implementation. When the health care worker data were analysed separately, there was no significant change in growth rates before and after the hinge day (S1 File) and there is no reason to believe that mask usage changed in the health care setting during the study period. These lack of changes in the health care worker cases supports the case that the changes observed in the general population are due to increased mask wearing in that population and not some general environmental factor.

We did not have access to daily case numbers for residents of aged care facilities over the whole of the analysis period, but the available data suggests that the cases in this group were growing rapidly during this time. Again, staff in aged care facilities were using PPE, including masks, extensively prior to masks becoming mandatory in the general community. This would suggest that the introduction of the mandatory mask policy would have less of an impact in this setting, given masks were already in use, hence leading to an underestimate of the overall impact of masks. Availability of more detailed case numbers over the whole Stage 3 and Stage 3+ masks period would allow this possibility to be tested rigorously. In addition, our analysis did not take account of transmission of COVID-19 in settings where mask use was not required, such as households or some workplaces. Given very high transmission within households [30], again, this would lead to an underestimation of the impact of the widespread use of masks.

## Conclusions

Our results provide strong evidence to support that the policy of mandatory face masks was effective in reducing COVID-19 cases in a Melbourne with restrictions already in place, accelerating the reduction of SARS-CoV-2 transmission. For the reasons outlined above, we have probably underestimated the impact of masks in the community more broadly. Our work also indicates a high level of compliance with the Victorian Government’s policy, and the rapid increase in mask use following a mandate. Face masks, whilst somewhat inconvenient for the individual user, are less likely to have unintended negative impacts on the broader community than policies restricting movement, social engagement and the operations of business, schools and childcare. While the observe change in growth rates were specific for the situation in Melbourne in mid 2020, never-the -less, our work strongly supports consideration of the use of face masks in other settings in Australia and globally to reduce community transmission of SARS-CoV-2.

## Supporting information

S1 File Regression goodness-of fit tests, analysis of confounding variables and calculation of *R*_eff_. (DOCX)

S1 Data Metadata detailing time, date and location of the photographs used enumerating mask usage. (XLSX)
