# Expression of Concern: The introduction of a mandatory mask policy was associated with significantly reduced COVID-19 cases in a major metropolitan city

**DOI:** 10.1371/journal.pone.0284939

**Published:** 2023-04-21

**Authors:** The PLOS ONE Editors

After publication of this article [1], readers raised a number of concerns, including about the methodology, the limitations of the study design, and whether the conclusions are fully supported. The *PLOS ONE* Editors consulted with two members of the editorial board and a statistical advisor who advised that the study design is associated with a number of weaknesses that are discussed in the article, and which are unavoidable because of ethical issues that would be associated with a randomized controlled trial in the context of a pandemic, but that there were also additional weaknesses. The authors have provided further discussion of these issues upon editorial follow up.

The following three sections summarize the concerns that were raised and the input received from the consulting experts and the authors.

## 1. Concerns raised about the photographic observation of mask usage

Changes in mask-wearing behavior were assessed by analyzing 44 photographs from a digital newspaper archive of public locations in Melbourne captured in the periods before and after introduction of a mask-wearing mandate. It is unclear whether such images are representative of mask usage in the population. Additionally, the data in the Supporting Information file S1 Data of [1] indicate that samples for the different periods were captured at different locations and at different times of day which may introduce bias (the photographs in the “pre mask” period were captured between 14.21 and 16.06, while the photographs in the “mandatory mask wearing period” were captured between 08.27 and 12.33).

Expert advice provided to the *PLOS ONE* Editors in follow up indicated that the sample of photographs taken for purposes other than this study may not be representative, particularly in view of the timing and location differences; however, this was considered a valid approach to acquire data about mask usage. It was also noted that the sample size of photographs is small; however, it was noted that the effect size was marked, supporting a correlation between the mask mandate and an increase in mask wearing.

## 2. Concerns raised about the Survey of COVID-19 Responses to Understand Behaviour (SCRUB) project—analysis of mask use

Self-reported mask use data were taken from round 6 of the Survey of COVID-19 Responses to Understand Behaviour (SCRUB) study (S1 File of [1]). The number of responders to the question about mask-wearing over the preceding 7 days was lower in the period after introduction of the mask-wearing mandate (n = 104 for Victoria; n = 146 for the other four states combined) compared to the period before the mandate (n = 704 for Victoria; n = 400 for the other four states combined). The sample size for this data set is small and may not be representative of the population. There were no responders outside of Victoria on the last two dates included (25^th^-26^th^ July).

The *PLOS ONE* Editors received expert input that the much smaller sample size for the SCRUB survey data in the post-mandate period reduces the probability of the post-mask mandate estimates being fair estimates of true mask use.

Regarding both the photographic and survey-based studies of mask usage, the authors indicated that the sample sizes were not inappropriately small for the purpose, noting that the high-powered interrupted time series study found a highly statistically significant change in the growth rate of the epidemic that coincided with the mask mandate. They further stated that follow-up tests of mask usage change would need to establish no change in mask usage in order to reject the hypothesis that the mask mandate could have caused the change in COVID growth rate, and that while some bias cannot be ruled out, both sources of mask usage data (photographs and surveys) showed a highly statistically significant change in usage.

## 3. Concerns raised about the assessment of confounders and interpretation

The study design could not exclude the possibility of contributions from unmeasured confounding variables, including the implementation of a curfew and movement restrictions on 2^nd^ August 2020 and closure of childcare facilities, schools, and non-essential businesses on 5^th^ August 2020. In view of the study design limitations, conclusions about causation cannot be drawn.

The consulted experts advised *PLOS ONE* Editors that the article [1] included a substantial discussion of methodological limitations and analyses to examine the influence of some potential confounders, though it was noted that this may not have been comprehensive; unmeasured confounding or the potential effect of saturation of the highly-exposed pool of persons could have interfered with causal inference, such that the data obtained are not adequate to fully support a conclusion that mask wearing is responsible for the reduction in COVID cases.

The authors stated that changes in diagnoses from the policies announced on the 2nd and 5th of August 2020 are highly unlikely to have had an impact on this analysis, explaining that with a 4 day incubation period, further delays before an infected person was symptomatic and sought a PCR test, then further delays while the PCR tests were being performed and results transmitted to the health authorities, it is not possible for changes resulting from the first day of curfew on the 3^rd^ August to have been reflected in changes in reported cases before the last day of data collection on the 10th August and even less likely for changes resulting from new restrictions on the 5^th^ August. It was additionally noted that the regression analysis uses the reported cases over the whole period from the 31^st^ July to the 10^th^ August, and the analysis of the residuals (S1 File of [1]) shows no significant deviation from the best fit regression line after the 3^rd^ or 5^th^ August.

The authors noted that at the time of the change in growth rate (31^st^ July 2020), the epidemic reached a peak in daily diagnoses of 686 in a city of approximately 4 million people. The authors state that, given the experiences from other settings, it is implausible that saturation of infection could have occurred, noting that in particular Victoria had a subsequent peak of 46,995 daily diagnoses in January 2021, with >95% of the 16+ years population vaccinated.

The authors note that the interrupted time series is subject to errors if factors other than the intervention happen to coincidentally change the output measure. It was noted that the study examined a series of potential confounding factors and found none that falsified the simplest hypothesis that mask usage decreases the transmission of COVID; however, the authors note it is important to emphasize that this study did not try to find a correlation between mask usage and the reduction in the growth constant. The first subheading of the Results section inaccurately refers to association of masks with a change in epidemic growth rate. The correct subheading is Association of a mask mandate with a change in epidemic growth rate.

The authors further note that the analysis is specific for a single population, and extrapolation of results to another population needs to be done with care.

The authors have indicated that they do not view the limitations discussed as significant and have stated that they stand by the published conclusions of the article.

According to the cumulative expert input received by the *PLOS ONE* Editors, the results of the published study [1] contribute to the field of mask evaluation research, provided results are not overinterpreted and limitations are acknowledged. The *PLOS ONE* Editors felt that the conclusions, including those that imply causation, a direct correlation between COVID-19 cases and mask mandates, and the ability of masks for controlling epidemics, were not suitably tempered in light of the limitations of the study design. The *PLOS ONE* Editors issue this Expression of Concern to inform readers about the above considerations regarding study design and interpretation of the results.

The authors provide the following additional clarifications and corrections:

The 10^th^ author’s name is spelled incorrectly. The correct spelling is Chandini Raina MacIntyre.

In the Methods section under the heading “Photographic observation of mask use”, in the second sentence there is an error in the reported time period of photographic data gathering. The correct sentence is:

To ensure we captured all images (not just those published), the librarian/digital archivist from the newspaper reviewed consecutive photos in their archive that were taken between July 10 and August 2, 2020 (13 days before and 11 days after mandatory mask policy introduction).

There is an error in the first sentence of the Results section, which incorrectly specifies that cases from Melbourne were analyzed. The correct sentence is:

There were 11,714 cases reported in Victoria between 10th July and 10th August inclusive, with daily cases increasing from 143 cases on 10th July 10 to a peak of 686 cases on 5th August, before declining to 310 cases on 10th August.

In the Results section under the heading “Photographic observation of mask use”, the first sentence incorrectly omits mention of unpublished photographs, which constituted the majority of the sample. The correct sentence is:

We analysed 44 published and unpublished photographic images taken between July 10 and August 2, 2020.

There is an error in the right-hand panel of Fig 2, which shows 0% mask wearing in public in the past 7 days for all regions aside from Victoria on 25^th^ -26^th^ July. No responses were recorded for individuals outside of Victoria at these two time points; as such these two data points are incorrect. Here the authors provide a corrected Fig 2. Hi

**Fig 2:**
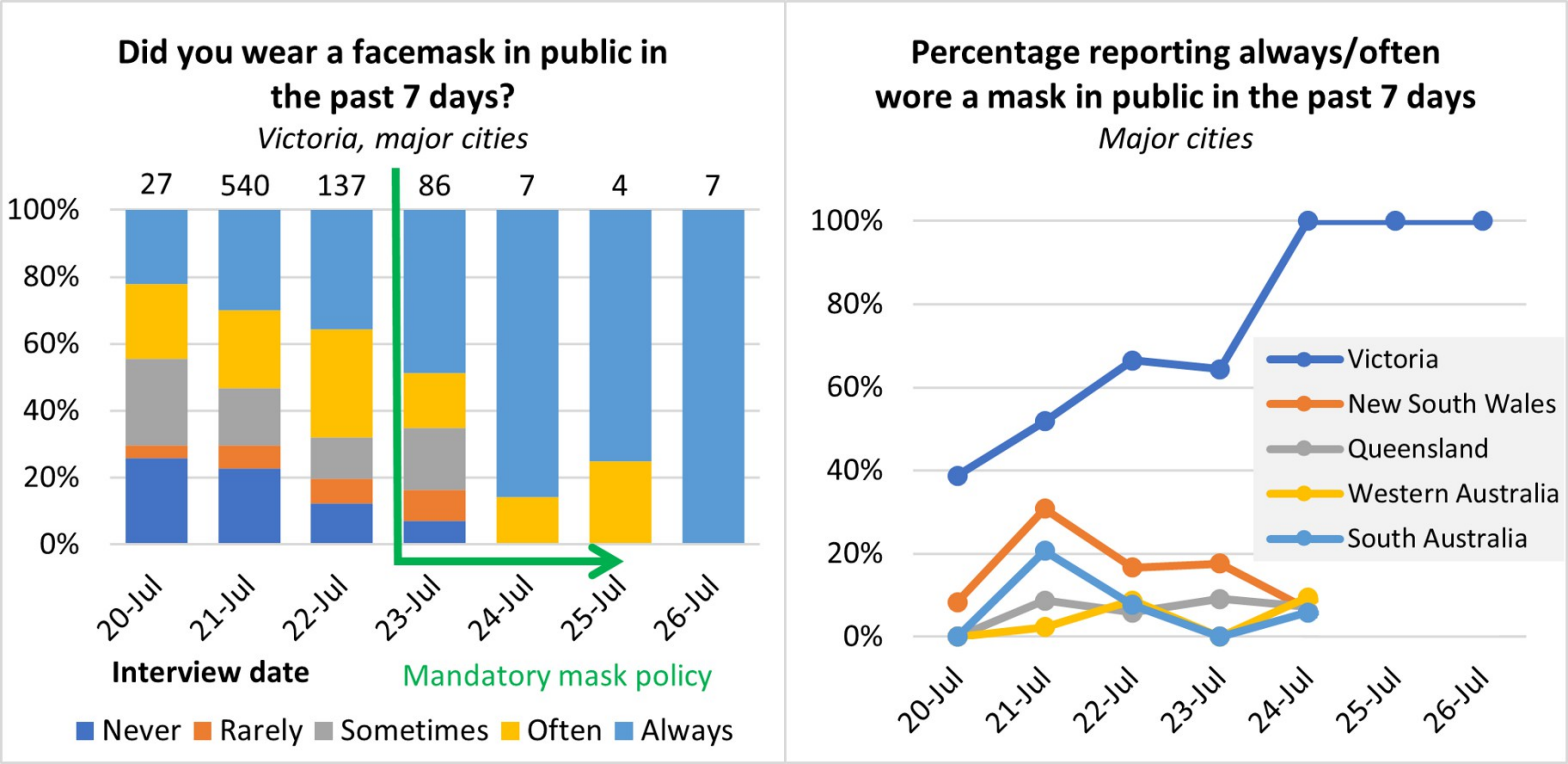
Results from the SCRUB survey. Left: for Round 6 respondents (20–26 July) in Victorian Stage 4 restriction areas, reported frequency of mask use in the past 7 days. Numbers on top of bars represent total respondents. Right: the percentage of respondents who reported always or often wearing a mask over time, Victorian Stage 4 restriction areas and compared to major cities in other states. The number of respondents varied from survey to survey and state to state but averaged 38.6 for each state and time point and the details are listed in S1 File.

Reference [28] of the original article is the uncorrected pre-print version of reference [1] cited in the original article.

A revised version of the Supporting Information S1 File is provided below, which has been updated to include the underlying data for the left-hand panel of Fig 2 (Table S5 in S1 File) and the underlying data used in the primary and secondary regression analyses (Table S3 in S1 File).

## Supporting information

S1 File Regression goodness-of fit tests, analysis of confounding variables, calculation of Reff, and underlying data. (DOCX)

## References

[1] Scott N, Saul A, Spelman T, Stoove M, Pedrana A, Saeri A, et al. (2021) The introduction of a mandatory mask policy was associated with significantly reduced COVID-19 cases in a major metropolitan city. PLoS ONE 16(7): e0253510. 10.1371/journal.pone.0253510 34288910 PMC8294480

