# Supplementary material for: The introduction of a mandatory mask policy was associated with significantly reduced COVID-19 cases in a major metropolitan city
Source: PLoS One. 2021 Jul 21;16(7):e0253510. doi: 10.1371/journal.pone.0253510 (PMC8294480; doi:10.1371/journal.pone.0253510)
Supplement: S1 File — (DOCX) [file pone.0253510.s001.docx]

1. **Goodness of fit tests for regression analysis**

This supplement describes the tests undertaken on the regression analysis shown in Figure 1 of the main paper

In a valid multiple linear regression, it is expected that the residuals meet the following criteria

- **Linearity**: The*ε_i_*have mean of 0
- **Independence**: The*ε_i_* are independent
- **Normality**: The *ε_i_* are normally distributed
- **Homogeneity of variances**: The*ε_i_*have the same variance *σ*^2^

We primarily test using the Studentized residuals *ε_i_* since raw residuals are not expected to be completely independent but provide some test results with the raw residuals

**Linearity**

|  | Mean |
| --- | --- |
| Raw Residuals | 3.6E-16 |
| Studentized residuals | 1.5E-15 |

Expect zero. Pass

**Independence**

*Durban-Watson Test*

| Test value negative autocorrelation | 2.4059 |
| --- | --- |
| Test value postitive autocorrelation | 1.5941 |
| Critical upper limit for α = 0.05 | 1.5736 |
|  |  |

Test values are both greater than the critical upper limit so no positive or negative autocorrelation is detected. Therefore there is no evidence for lack of independence.

*Graphical display*


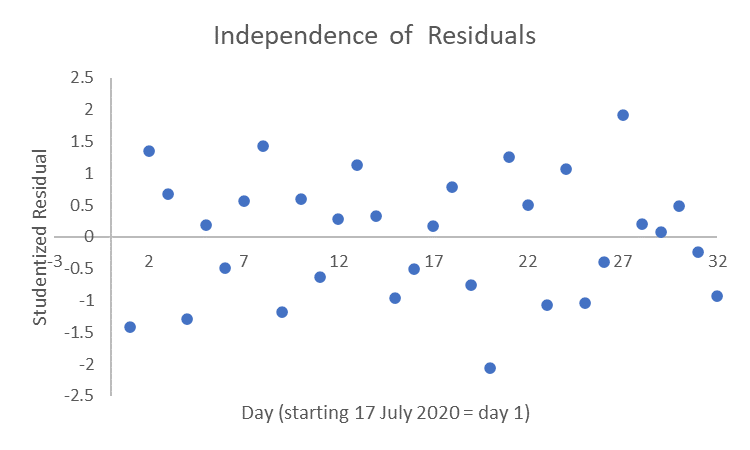


Fig S1. Plot of Studentized residuals *versus* day of the study. The Hinge day was day 22.

**Normality**

*Shapiro-Wilk test*

|  | Raw residual | Studentized residual |
| --- | --- | --- |
| W-stat | 0.9811 | 0.9846 |
| p-value | 0.8321 | 0.9164 |

Test passes for both raw and studentized residuals (p>0.05)

*Graphical QQ Plot*


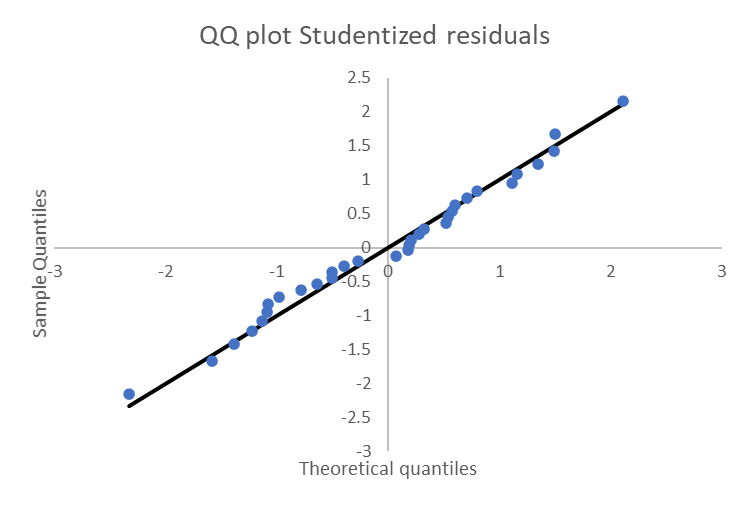


Fig S2. QQ (Quantile-Quantile) plot of the ordered Studentized residuals vs a the corresponding quantiles of a normal distribution. Black line indicates the position of a perfectly normally distributed Studentized residuals.

**Homogeneity of Variances**

*Test for Heteroskedasticity with Breusch-Pagan test*

| Number of Days | 32 |
| --- | --- |
| No. of Independent variables | 2 |
| LM statistic | 0.668576 |
| Degrees of Freedom | 2 |
| p-value | 0.715848 |

Pass criteria: p value >0.05. Pass

**Other test**

Regression was tested for the infludence of outliers using Cook’s D test. As shown below, the maximum Cook’s D value was 0.16 on day 20 (29 July 2020). As this is much less than 1, there are no outliers with a significant impact on the regression


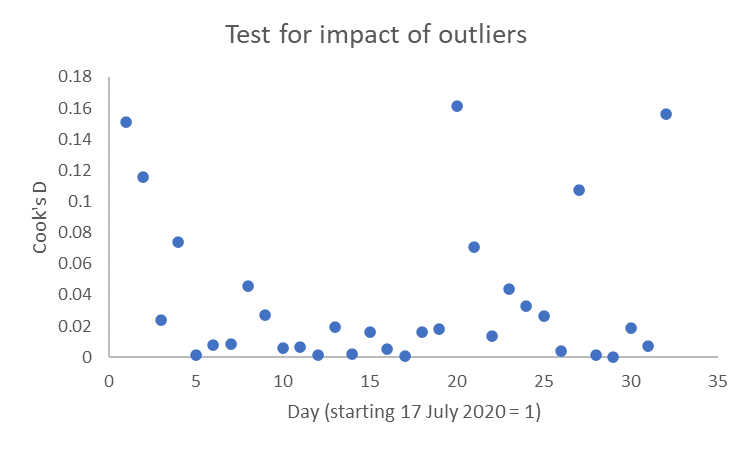


**Hinge day sensitivity analysis**

Table S1 shows the outcomes of linear spline models with the hinge on different days. For the main analysis, the hinge day for the main analysis was estimated to be 8 days following the introduction of masks, 31^st^ July

**Table S1. Sensitivity analysis for hinge day in the linear spline model.** Estimate for the change in slope parameter for models that assume different transition dates

| **Day mask impact observed** | **Estimate** | **Std. Error** | **t value** | **Pr(>\|t\|)** | **Adjusted R^2^ for overall model** |
| --- | --- | --- | --- | --- | --- |
| 29-Jul | -0.052 | 0.021 | -2.619 | 0.014 | 0.530 |
| 30-Jul | -0.060 | 0.021 | -2.863 | 0.008 | 0.547 |
| ***31-Jul*** | ***-0.065*** | ***0.022*** | ***-2.953*** | ***0.006*** | ***0.554*** |
| 1-Aug | -0.070 | 0.024 | -2.964 | 0.007 | 0.552 |
| 2-Aug | -0.080 | 0.0264 | -2.744 | 0.010 | 0.558 |

1. **Tests of potentially confounding variables**

Table S2 shows the secondary regression analyses conducted in this study to assess potentially confounding variables.

**Table S2. Secondary regression analyses**

|  | Estimate | Std. Error | t value | Pr(>\|t\|) |
| --- | --- | --- | --- | --- |
| ***Daily cases less Rural cases*** | | | | |
| Intercept | 5.330 | 0.099 | 53.669 | 0.000 |
| Slope estimate, pre-mask | 0.039 | 0.007 | 5.309 | 0.000 |
| Slope estimate, post-masks | -0.023 | 0.022 | -1.294 | 0.206 |
| **Change in slope, introduction of mandatory masks** | **-0.063** | **0.0293** | **-2.601** | **0.015** |
|  |  |  |  |  |
| ***Daily cases less HCW cases*** |  |  |  |  |
| Intercept | 5.249 | 0.113 | 46.537 | 0.000 |
| Slope estimate, pre-mask | 0.036 | 0.008 | 4.242 | 0.000 |
| Slope estimate, post-masks | -0.024 | 0.017 | -1.184 | 0.246 |
| **Change in slope, introduction of mandatory masks** | **-0.060** | **0.0274** | **-2.190** | **0.037** |
|  |  |  |  |  |
| ***Daily positive test ratio*** |  |  |  |  |
| Intercept | -4.971 | 0.118 | -41.908 | 0.000 |
| Slope estimate, pre-mask | 0.0534 | 0.009 | 5.975 | 0.000 |
| Slope estimate, post-masks | -0.0016 | 0.0215 | -0.0747 | 0.941 |
| **Change in slope, introduction of mandatory masks** | **-0.055** | **0.0347** | **-1.586** | **0.124** |
|  |  |  |  |  |
| ***Daily rural cases*** |  |  |  |  |
| Intercept | 0.946 | 0.262 | 3.609 | 0.001 |
| Slope estimate, pre-mask | 0.117 | 0.020 | 5.930 | 0.000 |
| Slope estimate, post-masks | -0.015 | 0.047 | -0.311 | 0.758 |
| **Change in slope, introduction of mandatory masks** | **-0.132** | **0.0637** | **-2.069** | **0.0475** |
|  |  |  |  |  |
| ***Daily HCW cases*** |  |  |  |  |
| Intercept | 2.409 | 0.111 | 21.633 | 0.000 |
| Slope estimate, pre-mask | 0.117 | 0.020 | 5.930 | 0.000 |
| Slope estimate, post-masks | 0.092 | 0.008 | 10.961 | 0.000 |
| **Change in slope, introduction of mandatory masks** | **-0.025** | **0.027** | **-0.932** | **0.359** |
|  |  |  |  |  |
| **Original regression including tests and mobility variables** |  |  |  |  |
| Intercept | 5.591 | 0.515 | 10.847 | 0.000 |
| Slope estimate, pre-mask | 0.041 | 0.008 | 5.369 | 0.000 |
| Slope estimate, post-masks | -0.036 | 0.024 | -1.526 | 0.139 |
| Tests coefficient | 0.000 | 0.000 | 0.930 | 0.360 |
| Mobility coefficient | -0.034 | 0.031 | -1.091 | 0.285 |
| **Change in slope, introduction of mandatory masks** | **-0.078** | **0.026** | **-2.981** | **0.006** |

**Changes in daily temperature**
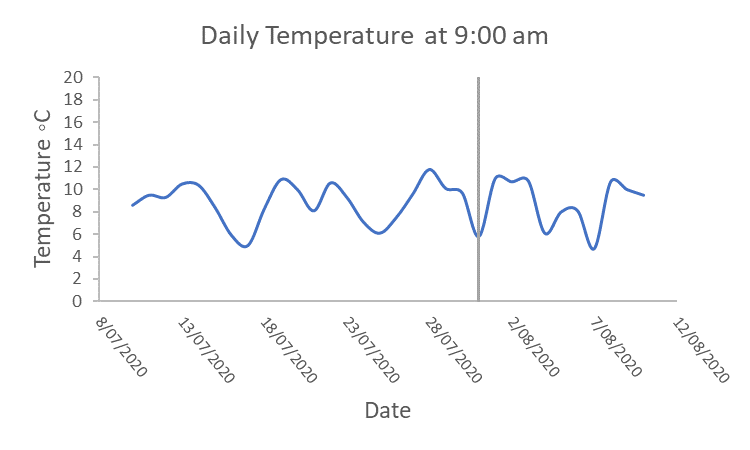


Daily temperature recorded at Olympic Park, Melbourne pre-and post-mask introduction. The hinge day for mask introduction was the 31 July (grey vertical line). Data suppled by the Australian Bureau of Meteorology

1. **Detailed responses to the SCRUBS face mask survey**

|  |  | Responders who often or always used mask | | | | |  | Total number of responders | | | | |
| --- | --- | --- | --- | --- | --- | --- | --- | --- | --- | --- | --- | --- |
| Survey start date |  | Victoria | New South Wales | Queensland | Western Australia | South Australia |  | Victoria | New South Wales | Queensland | Western Australia | South Australia |
| 20/07/2020 |  | 12 | 1 | 0 | 0 | 0 |  | 27 | 12 | 2 | 2 | 1 |
| 21/07/2020 |  | 288 | 40 | 7 | 1 | 6 |  | 540 | 126 | 75 | 40 | 26 |
| 22/07/2020 |  | 93 | 9 | 2 | 2 | 1 |  | 137 | 50 | 32 | 22 | 12 |
| 23/07/2020 |  | 56 | 6 | 1 | 0 | 0 |  | 86 | 32 | 11 | 4 | 7 |
| 24/07/2020 |  | 7 | 3 | 1 | 2 | 1 |  | 7 | 44 | 14 | 20 | 14 |
| 25/07/2020 |  | 4 | 0 | 0 | 0 | 0 |  | 4 | 0 | 0 | 0 | 0 |
| 26/07/2020 |  | 7 | 0 | 0 | 0 | 0 |  | 7 | 0 | 0 | 0 | 0 |
|  |  |  |  |  |  |  |  |  |  |  |  |  |

1. **Calculations of *R_eff_***

From Wallinga and Lipsitch, for a serial interval with a normal distritribution with mean (µ), standard deviarion (σ), if the epidemic is growing exponentially with an exponential growth rate k, the effective reproduction ratio R_eff_ can be calculated as

$$R_{eff}=\exp\left( k\mu-\frac{1}{2}k^{2}\sigma^{2} \right)$$

While for a serial interval with a gamma distritribution, if α and β are the shape and rate parameters respectively, µ and σ are the corresponding mean and standard deviarion respectively, k is the exponential growth rate, and g(x) is the probability density function of the gamma distribution, then R_eff_ can be calculated as

| $\frac{1}{R_{eff}}=\int_{x=0}^{\infty} e^{-kx}g\left( x \right)dx$ |  |
| --- | --- |
| $=\int_{x=0}^{\infty} e^{-kx}\frac{\left( \beta^{\alpha}x^{\alpha-1}e^{-\beta x} \right)}{\Gamma\left( \alpha\right)}dx$ | (definition of Gamma probability density function, where  Γ(α) is the Gamma function $\Gamma\left( \alpha\right)=\int_{0}^{\infty} x^{\alpha-1}e^{-x}dx$) |
| $=\frac{\beta^{\alpha}}{\Gamma\left( \alpha\right)}\int_{x=0}^{\infty} e^{-\left( k+\beta\right)x}x^{\alpha-1}dx$ |  |
| $=\frac{\beta^{\alpha}}{\Gamma\left( \alpha\right)}\frac{\Gamma\left( \alpha\right)}{{(k+\beta)}^{\alpha}}$ | (Since this was the Laplace transform of x^α-1^) |
| $=\frac{\beta^{\alpha}}{\left( k+\beta\right)^{\alpha}}$ |  |
| $R_{eff}=\left( \frac{k\sigma^{2}}{\mu}+1 \right)^{\frac{\mu^{2}}{\sigma^{2}}}$ |  |

1. **References**

All tests were performed using the Excel routines available from

Zaiontz, C. 2020 Real Statistics Using Excel. Version 7.3.3 <https://www.real-statistics.com/>

Wallinga, J., & Lipsitch, M. (2007). How generation intervals shape the relationship between growth rates and reproductive numbers. Proceedings of the Royal Society B: Biological Sciences, 274(1609), 599-604.
