# Supplementary material for: Expression of Concern: The introduction of a mandatory mask policy was associated with significantly reduced COVID-19 cases in a major metropolitan city
Source: PLoS One. 2023 Apr 21;18(4):e0284939. doi: 10.1371/journal.pone.0284939 (PMC10120923; doi:10.1371/journal.pone.0284939)
Supplement: S1 File — (DOCX) [file pone.0284939.s001.docx]

1. **Goodness of fit tests for regression analysis**

This supplement describes the tests undertaken on the regression analysis shown in Figure 1 of the main paper.

In a valid multiple linear regression, it is expected that the residuals meet the following criteria:

- **Linearity**: The*ε_i_*have mean of 0
- **Independence**: The*ε_i_* are independent
- **Normality**: The *ε_i_* are normally distributed
- **Homogeneity of variances**: The*ε_i_*have the same variance *σ*^2^

We primarily test using the Studentized residuals *ε_i_* since raw residuals are not expected to be completely independent but provide some test results with the raw residuals.

**Linearity**

|  | Mean |
| --- | --- |
| Raw Residuals | 3.6E-16 |
| Studentized residuals | 1.5E-15 |

Expect zero. Pass

**Independence**

*Durban-Watson Test*

| Test value negative autocorrelation | 2.4059 |
| --- | --- |
| Test value postitive autocorrelation | 1.5941 |
| Critical upper limit for α = 0.05 | 1.5736 |
|  |  |

Test values are both greater than the critical upper limit so no positive or negative autocorrelation is detected. Therefore there is no evidence for lack of independence.

*Graphical display*


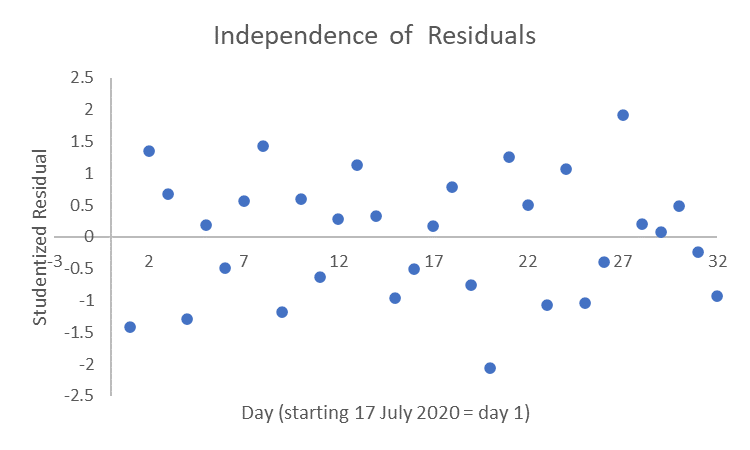


Fig S1. Plot of Studentized residuals *versus* day of the study. The Hinge day was day 22.

**Normality**

*Shapiro-Wilk test*

|  | Raw residual | Studentized residual |
| --- | --- | --- |
| W-stat | 0.9811 | 0.9846 |
| p-value | 0.8321 | 0.9164 |

Test passes for both raw and studentized residuals (p>0.05)

*Graphical QQ Plot*


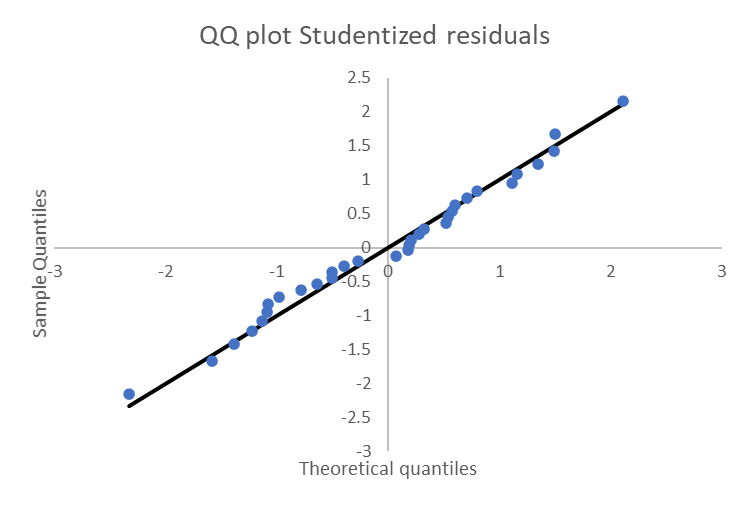


Fig S2. QQ (Quantile-Quantile) plot of the ordered Studentized residuals vs a the corresponding quantiles of a normal distribution. Black line indicates the position of a perfectly normally distributed Studentized residuals.

**Homogeneity of Variances**

*Test for Heteroskedasticity with Breusch-Pagan test*

| Number of Days | 32 |
| --- | --- |
| No. of Independent variables | 2 |
| LM statistic | 0.668576 |
| Degrees of Freedom | 2 |
| p-value | 0.715848 |

Pass criteria: p value >0.05. Pass

**Other test**

Regression was tested for the infludence of outliers using Cook’s D test. As shown below, the maximum Cook’s D value was 0.16 on day 20 (29 July 2020). As this is much less than 1, there are no outliers with a significant impact on the regression.


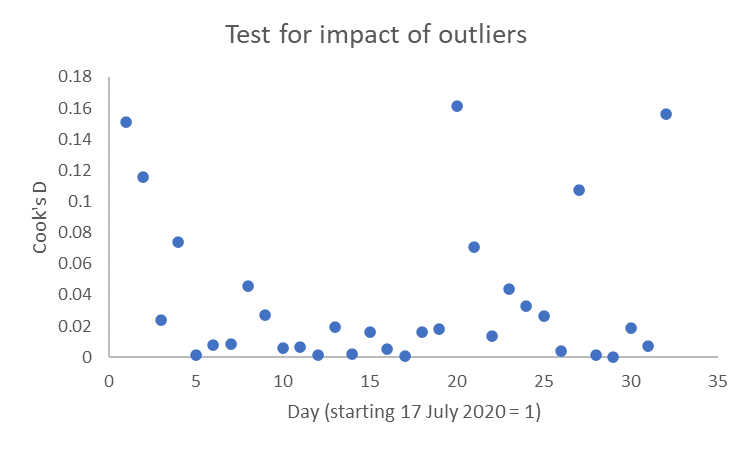


All tests were performed using the Excel routines available from [1].

**Hinge day sensitivity analysis**

Table S1 shows the outcomes of linear spline models with the hinge on different days. For the main analysis, the hinge day for the main analysis was estimated to be 8 days following the introduction of masks, 31^st^ July.

**Table S1. Sensitivity analysis for hinge day in the linear spline model.** Estimate for the change in slope parameter for models that assume different transition dates.

| **Day mask impact observed** | **Estimate** | **Std. Error** | **t value** | **Pr(>\|t\|)** | **Adjusted R^2^ for overall model** |
| --- | --- | --- | --- | --- | --- |
| 29-Jul | -0.052 | 0.021 | -2.619 | 0.014 | 0.530 |
| 30-Jul | -0.060 | 0.021 | -2.863 | 0.008 | 0.547 |
| ***31-Jul*** | ***-0.065*** | ***0.022*** | ***-2.953*** | ***0.006*** | ***0.554*** |
| 1-Aug | -0.070 | 0.024 | -2.964 | 0.007 | 0.552 |
| 2-Aug | -0.080 | 0.0264 | -2.744 | 0.010 | 0.558 |

**Tests of potentially confounding variables**

**Table S2. Secondary regression analyses**

|  | Estimate | Std. Error | t value | Pr(>\|t\|) |
| --- | --- | --- | --- | --- |
| ***Daily cases less Rural cases*** | | | | |
| Intercept | 5.330 | 0.099 | 53.669 | 0.000 |
| Slope estimate, pre-mask | 0.039 | 0.007 | 5.309 | 0.000 |
| Slope estimate, post-masks | -0.023 | 0.022 | -1.294 | 0.206 |
| **Change in slope, introduction of mandatory masks** | **-0.063** | **0.0293** | **-2.601** | **0.015** |
|  |  |  |  |  |
| ***Daily cases less HCW cases*** |  |  |  |  |
| Intercept | 5.249 | 0.113 | 46.537 | 0.000 |
| Slope estimate, pre-mask | 0.036 | 0.008 | 4.242 | 0.000 |
| Slope estimate, post-masks | -0.024 | 0.017 | -1.184 | 0.246 |
| **Change in slope, introduction of mandatory masks** | **-0.060** | **0.0274** | **-2.190** | **0.037** |
|  |  |  |  |  |
| ***Daily positive test ratio*** |  |  |  |  |
| Intercept | -4.971 | 0.118 | -41.908 | 0.000 |
| Slope estimate, pre-mask | 0.0534 | 0.009 | 5.975 | 0.000 |
| Slope estimate, post-masks | -0.0016 | 0.0215 | -0.0747 | 0.941 |
| **Change in slope, introduction of mandatory masks** | **-0.055** | **0.0347** | **-1.586** | **0.124** |
|  |  |  |  |  |
| ***Daily rural cases*** |  |  |  |  |
| Intercept | 0.946 | 0.262 | 3.609 | 0.001 |
| Slope estimate, pre-mask | 0.117 | 0.020 | 5.930 | 0.000 |
| Slope estimate, post-masks | -0.015 | 0.047 | -0.311 | 0.758 |
| **Change in slope, introduction of mandatory masks** | **-0.132** | **0.0637** | **-2.069** | **0.0475** |
|  |  |  |  |  |
| ***Daily HCW cases*** |  |  |  |  |
| Intercept | 2.409 | 0.111 | 21.633 | 0.000 |
| Slope estimate, pre-mask | 0.117 | 0.020 | 5.930 | 0.000 |
| Slope estimate, post-masks | 0.092 | 0.008 | 10.961 | 0.000 |
| **Change in slope, introduction of mandatory masks** | **-0.025** | **0.027** | **-0.932** | **0.359** |
|  |  |  |  |  |
| **Original regression including tests and mobility variables** |  |  |  |  |
| Intercept | 5.591 | 0.515 | 10.847 | 0.000 |
| Slope estimate, pre-mask | 0.041 | 0.008 | 5.369 | 0.000 |
| Slope estimate, post-masks | -0.036 | 0.024 | -1.526 | 0.139 |
| Tests coefficient | 0.000 | 0.000 | 0.930 | 0.360 |
| Mobility coefficient | -0.034 | 0.031 | -1.091 | 0.285 |
| **Change in slope, introduction of mandatory masks** | **-0.078** | **0.026** | **-2.981** | **0.006** |

**Table S3.** Daily total and rural Covid Cases, number of PCR tests and mobility data used for analysis in Table S2.

| Date | Total daily cases | Rural cases | HCW cases | Daily PCR tests | % mobility |
| --- | --- | --- | --- | --- | --- |
| 10/07/2020 | 143 | 1 | 14 | 37,588 | 14 |
| 11/07/2020 | 289 | 4 | 13 | 27,169 | 13 |
| 12/07/2020 | 256 | 8 | 11 | 30,195 | 15 |
| 13/07/2020 | 167 | 5 | 17 | 22,943 | 16 |
| 14/07/2020 | 248 | 5 | 12 | 21,995 | 16 |
| 15/07/2020 | 220 | 5 | 11 | 27,040 | 14 |
| 16/07/2020 | 295 | 5 | 18 | 28,607 | 15 |
| 17/07/2020 | 379 | 1 | 19 | 24,409 | 14 |
| 18/07/2020 | 212 | 9 | 27 | 28,104 | 12 |
| 19/07/2020 | 338 | 11 | 20 | 26,674 | 13 |
| 20/07/2020 | 263 | 13 | 30 | 26,588 | 15 |
| 21/07/2020 | 341 | 33 | 48 | 29,464 | 13 |
| 22/07/2020 | 436 | 2 | 54 | 24,726 | 12 |
| 23/07/2020 | 375 | 14 | 29 | 27,151 | 12 |
| 24/07/2020 | 288 | 26 | 46 | 24,118 | 13 |
| 25/07/2020 | 335 | 13 | 63 | 20,313 | 13 |
| 26/07/2020 | 411 | 9 | 68 | 42,973 | 14 |
| 27/07/2020 | 495 | 11 | 48 | 17,588 | 13 |
| 28/07/2020 | 358 | 21 | 32 | 18,521 | 13 |
| 29/07/2020 | 273 | 16 | 82 | 18,077 | 13 |
| 30/07/2020 | 629 | 61 | 69 | 19,921 | 12 |
| 31/07/2020 | 549 | 29 | 65 | 33,826 | 12 |
| 1/08/2020 | 368 | 22 | 62 | 25,501 | 13 |
| 2/08/2020 | 600 | 37 | 50 | 32,000 | 16 |
| 3/08/2020 | 354 | 40 | 63 | 11,000 | 14 |
| 4/08/2020 | 404 | 23 | 114 | 21,000 | 12 |
| 5/08/2020 | 686 | 39 | 82 | 23,947 | 9 |
| 6/08/2020 | 445 | 30 | 75 | 25,000 | 9 |
| 7/08/2020 | 421 | 33 | 74 | 18,133 | 8 |
| 8/08/2020 | 454 | 24 | 75 | 18,133 | 8 |
| 9/08/2020 | 373 | 27 | 62 | 18,134 | 9 |
| 10/08/2020 | 310 | 19 | 54 | 19,398 | 9 |

The total daily cases time-series used for this analysis was extracted from the Victorian Department of Health and Human Services dataset at the time of the analysis (November 11, 2020), which listed daily reported cases by local government area and acquired source, which were then assigned to metropolitan and non-metropolitan areas as defined in the main paper (ref 3, main paper)[2]. Daily PCR tests and HCW data were from the Victorian Department of Health and Human services daily update[3]. Note that case numbers were also reported in the Victorian Government daily communications (e.g. press conferences, social media) but the number and location of cases assigned to each date could differ slightly as they were periodically revised. The date at which a case was reported in communications could also differ by approximately one day compared to the dataset, since the dataset represents the date of diagnosis, which would then be reported the following day. For consistency over the different datasets, the number of daily cases in the table above are taken from reference [2] but the date is the date reported (i.e. from reference [3]). Mobility data were from Citymapper. [4]

**Changes in daily temperature**
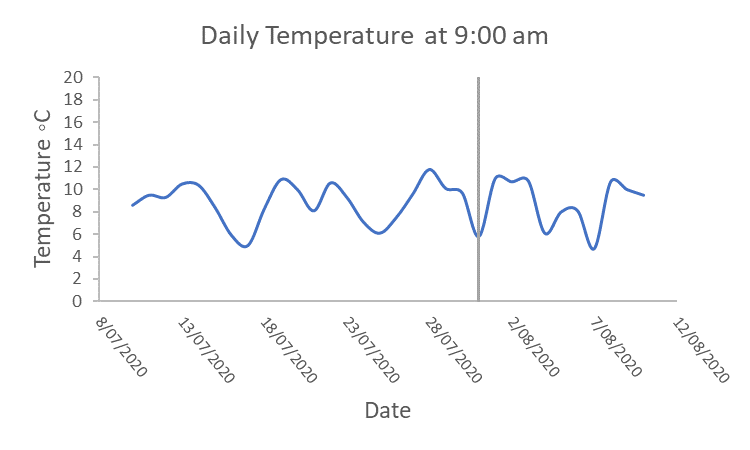


Daily temperature recorded at Olympic Park, Melbourne pre-and post-mask introduction. The hinge day for mask introduction was the 31 July (grey vertical line). Data supplied by the Australian Bureau of Meteorology

1. **Detailed responses to the SCRUBS face mask survey**

**Table S4. All state responses to survey**

|  |  | Responders who often or always used mask | | | | |  | Total number of responders | | | | |
| --- | --- | --- | --- | --- | --- | --- | --- | --- | --- | --- | --- | --- |
| Survey start date |  | Victoria | New South Wales | Queensland | Western Australia | South Australia |  | Victoria | New South Wales | Queensland | Western Australia | South Australia |
| 20/07/2020 |  | 12 | 1 | 0 | 0 | 0 |  | 27 | 12 | 2 | 2 | 1 |
| 21/07/2020 |  | 288 | 40 | 7 | 1 | 6 |  | 540 | 126 | 75 | 40 | 26 |
| 22/07/2020 |  | 93 | 9 | 2 | 2 | 1 |  | 137 | 50 | 32 | 22 | 12 |
| 23/07/2020 |  | 56 | 6 | 1 | 0 | 0 |  | 86 | 32 | 11 | 4 | 7 |
| 24/07/2020 |  | 7 | 3 | 1 | 2 | 1 |  | 7 | 44 | 14 | 20 | 14 |
| 25/07/2020 |  | 4 | 0 | 0 | 0 | 0 |  | 4 | 0 | 0 | 0 | 0 |
| 26/07/2020 |  | 7 | 0 | 0 | 0 | 0 |  | 7 | 0 | 0 | 0 | 0 |
|  |  |  |  |  |  |  |  |  |  |  |  |  |

**Table S5. Melbourne responses to survey.** Responses to people living in Melbourne to the question “In the past 7 days, how frequently have you taken the following actions?” and the specific item “Wear a face mask whenever in public” (response scale: Never, Rarely, Sometimes, Often, Always)

| Survey Date | Never | Rarely | Sometimes | Often | Always | Total |
| --- | --- | --- | --- | --- | --- | --- |
| 20/7/2020 | 7 | 1 | 7 | 6 | 6 | 27 |
| 21-Jul | 123 | 37 | 92 | 126 | 162 | 540 |
| 22-Jul | 17 | 10 | 17 | 44 | 49 | 137 |
| 23-Jul | 6 | 8 | 16 | 14 | 42 | 86 |
| 24-Jul | 0 | 0 | 0 | 1 | 6 | 7 |
| 25-Jul | 0 | 0 | 0 | 1 | 3 | 4 |
| 26-Jul | 0 | 0 | 0 | 0 | 7 | 7 |

1. **Calculations of *R_eff_***

From Wallinga and Lipsitch [5], for a serial interval with a normal distritribution with mean (µ), standard deviarion (σ), if the epidemic is growing exponentially with an exponential growth rate k, the effective reproduction ratio R_eff_ can be calculated as

$$R_{eff}=\exp\left( k\mu-\frac{1}{2}k^{2}\sigma^{2} \right)$$

While for a serial interval with a gamma distritribution, if α and β are the shape and rate parameters respectively, µ and σ are the corresponding mean and standard deviarion respectively, k is the exponential growth rate, and g(x) is the probability density function of the gamma distribution, then R_eff_ can be calculated as

| $\frac{1}{R_{eff}}=\int_{x=0}^{\infty} e^{-kx}g\left( x \right)dx$ |  |
| --- | --- |
| $=\int_{x=0}^{\infty} e^{-kx}\frac{\left( \beta^{\alpha}x^{\alpha-1}e^{-\beta x} \right)}{\Gamma\left( \alpha\right)}dx$ | (definition of Gamma probability density function, where  Γ(α) is the Gamma function $\Gamma\left( \alpha\right)=\int_{0}^{\infty} x^{\alpha-1}e^{-x}dx$) |
| $=\frac{\beta^{\alpha}}{\Gamma\left( \alpha\right)}\int_{x=0}^{\infty} e^{-\left( k+\beta\right)x}x^{\alpha-1}dx$ |  |
| $=\frac{\beta^{\alpha}}{\Gamma\left( \alpha\right)}\frac{\Gamma\left( \alpha\right)}{{(k+\beta)}^{\alpha}}$ | (Since this was the Laplace transform of x^α-1^) |
| $=\frac{\beta^{\alpha}}{\left( k+\beta\right)^{\alpha}}$ |  |
| $R_{eff}=\left( \frac{k\sigma^{2}}{\mu}+1 \right)^{\frac{\mu^{2}}{\sigma^{2}}}$ |  |

1. **References**

1. Zaiontz C. Real Statistics Using Excel. Version 7.3.3 2020 [cited 2022 October 13]. Available from: <https://www.real-statistics.com/>.

2. Victorian Department of Health and Human Services. All Victorian cases by local government area and acquired source (CSV) 2022 [updated 23 Sept 2022; cited 2022 27 Sept]. Available from: <https://www.dhhs.vic.gov.au/ncov-covid-cases-by-lga-source-csv>.

3. Victorian Department of Health and Human Services. Updates about the outbreak of the coronavirus disease (COVID-19). 2021 [updated October 28, 2021; cited 2022 October 10]. Available from: <https://www.dhhs.vic.gov.au/coronavirus/updates>.

4. CityMapper. Citymapper Mobility Index: Melbourne 2021 [updated 2021 September; cited 2022 October 10]. Available from: <https://citymapper.com/cmi/melbourne>.

5. Wallinga J, Lipsitch M. How generation intervals shape the relationship between growth rates and reproductive numbers. Proc Biol Sci. 2007;274(1609):599-604. doi: 10.1098/rspb.2006.3754. PubMed PMID: 17476782.
